# Effect of Internal Architecture on the Elasticity of Microgel Monolayers at the Air/Water Interface

**DOI:** 10.1021/acs.macromol.5c02434

**Published:** 2025-12-10

**Authors:** Wei Liu, Li Zhang, Zehua Han, He Cheng, Hailin Li, Xiangjun Gong, Hang Jiang, Yuwei Zhu, To Ngai

**Affiliations:** † The Key Laboratory of Synthetic and Biological Colloids, Ministry of Education & School of Chemical and Material Engineering, 66374 Jiangnan University, Wuxi 214122, China; ‡ Spallation Neutron Source Science Center (SNSSC), Dongguan 523803, China; § Faculty of Materials Science and Engineering, 26467 South China University of Technology, Guangzhou 510640, China; ∥ Department of Chemistry, 26451 The Chinese University of Hong Kong, Shatin, New Territories, Hong Kong 999077, China

## Abstract

Soft microgels can deform and adsorb at liquid interfaces, forming monolayers with tunable compressibility and elasticity. Their deformability—or softness—is governed by the internal architecture and inhomogeneity of the polymeric network. However, establishing a direct correlation between single-particle properties and their collective interfacial behaviors—such as self-assembly and mechanical response—remains a fundamental challenge. In this work, we employ core–shell microgels with controllable internal architectures to investigate how single-particle softness influences the elasticity of microgel monolayers at the air/water interface. Flory–Rehner analysis reveals that microgel softness is determined by internal architecture via elastic free energy, with mixing contributions nearly invariant, ultimately governing osmotic deswelling behavior. Application of a generalized Hertzian potential in the semidilute regime further reveals enhanced chain entanglement within the confined interfacial polymer layer. Moreover, by analyzing the relationship between interparticle interactions and nearest neighbor distance in the condensed regime, we quantify the interfacial elasticity of the monolayers. Our findings show that microgels with loosely or homogeneously cross-linked networks—rather than those with dense-core or dense-shell structures—yield higher elasticity. This counterintuitive result suggests that softer microgels can produce stiffer monolayers, which is further examined through the modulation of environmental and solution-related parameters.

## Graphical abstract

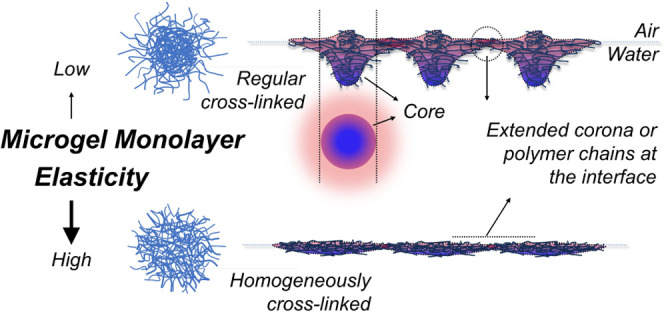

## Introduction

The polymer-to-colloid duality of microgels—cross-linked polymer networks swollen in good solvents—enables them to spread and flatten at liquid interfaces, imparting sophisticated self-assembly behavior and distinct interfacial mechanical responses.^1–3^ Their polymeric nature confers intrinsic softness and compressibility.^4^ At interfaces, microgels can spontaneously adsorb to form monolayers, thereby reducing interfacial tension and minimizing free energy. Unlike rigid hard spheres, soft microgels readily deform into irregular shape, often adopting the characteristic “fried-egg” morphology.^5,6^ This deformation arises from the interplay between surface tension and the flexibility of the polymer network. Dangling chains from the outer corona become highly stretched upon adsorption and can easily entangle with neighboring microgels when the monolayer is subjected to compression. This leads to a progressive transition in contact—from corona–corona to corona–core, and ultimately to core–core interactions—observable through compression isotherms and supported by both experimental and numerical evidence.^7–9^ While structural topology of single microgels at interfaces has been extensively studied,^6,10,11^ the collective interfacial behaviors remain poorly understood. Moreover, even less information has been disclosed about the elasticity of the entangled polymer chains within the monolayer, hindering a microscopic understanding of such two-dimensional (2D) soft systems.

Experimental approaches such as interfacial rheology, along with theoretical modeling, offer valuable strategies for probing mechanical responses in the 2D systems. Recent studies have investigated the rheological properties of poly­(*N*-isopropylacrylamide) (PNIPAM) microgel monolayers—widely regarded as an ideal model system for soft colloids—at fluid interfaces using both passive and active techniques.^12–16^ The elasticity of the microgel monolayer was shown to primarily correlate with packing fraction, interparticle distance, and aggregated structure.^12^ It was also related to individual particle deformation that originates from the internal architecture. For instance, the corona–corona contact regime, characterized by a highly ordered hexagonal lattice, exhibits a weak power-law dependence on shear rate.^13^ These findings highlight the pivotal role of microgel deformability, or softness, in determining the mechanical properties of the monolayers. Furthermore, complementary simulations have quantified the microgel interaction in two-body systems, revealing that interfacial elasticity is approximately 1 order of magnitude greater than that observed in bulk.^17^ This enhancement is attributed to reduced polymer chain mobility and increased stiffness, driven primarily by interfacial tension, which governs the polymer network’s response to external stress. Although direct computation of many–body interactions remains a challenge, the above efforts provide a first step toward a microscopic understanding of how softness of individual microgels affect the interfacial elasticity of the monolayers.

The intrinsic softness of microgels—and more broadly, of soft deformable entities—can be readily tuned via the cross-linking density, which in turn governs the internal architecture and inhomogeneity of the polymer network.^18–21^ Regular core–corona microgels, synthesized through batch precipitation polymerization, exhibit a compact core with a gradually decreasing cross-linking density toward the periphery in bulk solution. Upon adsorption at fluid interfaces, the fuzzy shell of these microgels stretches into a thin layer, while the core elastically deforms, protruding several nanometers into the air or oil phase yet remaining swollen in the aqueous phase.^6,11^ The thermal responsiveness and volume phase transition of the interfacial layer of PNIPAM microgels have been shown to be significantly suppressed due to interfacial confinement.^22^ Additionally, the influence of electrostatic charges on polyelectrolyte microgels has also been explored, revealing that electrostatic interactions affect the compressibility of the monolayer under confinement.^23^ Other types of microgels of particular interest are those with distinct internal architectures, such as homogeneously cross-linked, ultralow cross-linked (ULC),^24,25^ or hollow microgels with large internal cavities.^26–28^ These “softer” microgels exhibit complete flattening upon adsorption at fluid interfaces, behaving more like polymeric films than colloidal particles. Under compression, the interfacial packing and mechanical properties are initially dominated by the contact or entanglement of the outer dangling chains.^18^ Building on these observations, clearly, by tuning the single-microgel internal architecture, it is possible, in principle, to finely control the interfacial mechanics of microgel monolayers, motivating the present study.

In our last Letter, we experimentally investigated the role of intermacromolecular interactions in governing interfacial stress and self-assembly of pH-responsive microgels at the air/water interface.^29^ We highlighted the influence of interchain interactions under interfacial confinement on 2D phase transitions, with particular emphasis on spatiotemporal dynamics and energetic contributions. Building upon these findings, the present study offers a more comprehensive investigation of interchain interactions and elasticity of polymeric interfacial plane by systematically tuning the internal architecture of individual microgels. Leveraging a well-controlled synthetic protocol, we employ core–shell microgels with varied internal architectures as a model system to elucidate the impact of single-particle structure on collective behavior at the air/water interface. We begin by quantifying the structural parameters of the self-assembled structure using radial distribution functions. The intermicrogel potential of mean force is subsequently mapped in two dimensions by correlating surface pressure with interparticle distance. The resulting energy landscapes reveal distinct primary minima in both location and depth, reflecting the variable compressibility of the core–shell microgels. Furthermore, we demonstrate that surface pressure measurements obtained via Langmuir trough experiments can be directly correlated with nearest neighbor distances using a generalized Hertzian model, applicable within the semidilute regime. With these key parameters—interparticle potential and spacing—established, we quantify the elasticity of the entangled polymer chains within the interfacial plane under large compression using an effective elastic coefficient. Finally, we investigate how environmental variables—including temperature (thermal swelling/deswelling), ionic strength (electrostatic interactions), and solvent quality (polymer–solvent interactions)—modulate both the interparticle interactions and the mechanical properties of microgel monolayers.

Critically, this study aims to address a fundamental yet unresolved question in two-dimensional microgel systems: how the internal cross-linked architecture of polymer networks governs the softness of individual microgels. More specifically, we investigate how single-particle softness translate into collective interfacial mechanics, including the compressibility and elasticity of microgel monolayers. Resolving these issues will provide fundamental insight into the structure–mechanics relationship of soft polymeric systems under 2D confinement.

## Results and Discussion

### Internal Architecture Defines Microgel Softness

We begin by quantifying the softness of individual microgels with distinct internal architectures. To disentangle the role of internal architecture from overall cross-linking density in governing mechanical properties, we employ core–shell PNIPAM microgels with diameters of approximately 0.1–0.2 μm. All microgels used in this study contain 5 mol % of the cross-linker *N*,*N*′-methylenebis­(acrylamide) (BIS), ensuring a consistent total cross-linking density. By precisely controlling the synthetic procedure,^30^ particularly the timing of cross-linker introduction during polymerization, we produce three distinct architectures: (i) dense-core (DC) microgels with a highly cross-linked core, (ii) dense-shell (DS) microgels with a loosely cross-linked core and a dense periphery, and (iii) homogeneously cross-linked (HOMO) microgels, as illustrated in Figure 1a. The internal density profiles of these microgels are characterized using transmission electron microscopy (TEM), atomic force microscopy (AFM), and small-angle neutron scattering (SANS),^31,32^ as shown in Figure 1b,d,e. Notably, the fuzzy shell composed of dangling chains in DC microgels is directly visualized via TEM and corroborated by the fuzzy sphere model in SANS.^33^ In contrast, DS and HOMO microgels exhibit significantly reduced height profiles in the dry state, accompanied by pronounced lateral expansion or complete flattening (Figure 1d), indicating enhanced collapse of the polymer network. Further evidence of low-density architecture in DS and HOMO microgels is provided by the marked attenuation of scattering intensity in the low-*q* region of SANS data (Figure 1e), with HOMO microgels displaying the most pronounced softness. In addition, fluid AFM measurements mapping the 2D height profile and Young’s modulus of individual microgels, together with rheological tests of suspensions at identical effective volume fractions (ϕ_eff_ > 1, glassy regime), consistently demonstrate that HOMO microgels are the softest, DS microgels exhibit intermediate stiffness, and DC microgels are the stiffest among the three (Figure S1). Given the comparable chain flexibility of PNIPAM segments, nonuniform cross-linking density serves as a reliable proxy for the local softness within gel networks. Nevertheless, a universally accepted quantitative definition of softness for individual compressible microgels has not reached a consensus.

**Figure 1:**
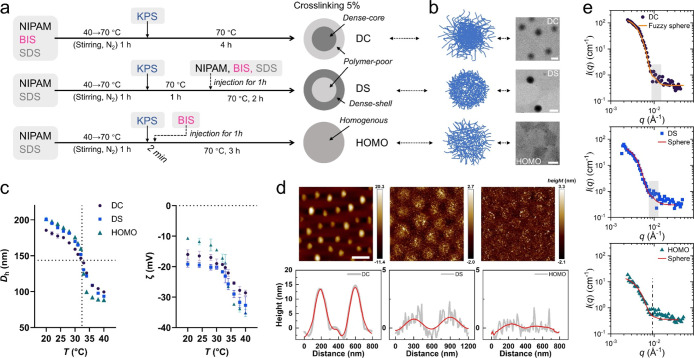
Microgel synthesis and characterizations. (a) Synthetic procedure for the dense-core (DC), dense-shell (DS), and homogeneously cross-linked (HOMO) microgels. Cross-linker (BIS) was introduced into the polymerization by a continuous injection at different timing. (b) Illustrates the cross-linking density of single microgels by transmission electron microscope (TEM). Scale bar: 100 nm (DC) and 500 nm (DS and HOMO). (c) Hydrodynamic diameter (*D*_h_) and zeta potential (ζ) at various temperatures. The dotted vertical line indicates the volume phase transition temperature (VPTT). (d) Shows the lateral morphology of microgels by atomic force microscope (AFM) at a dried state. Color bar donates the value of height in nm. Scale bar: 500 nm. Representative height profiles for two neighboring microgels are given (bottom). (e) Shows the small-angle neutron scattering (SANS) intensities *I*(*q*) versus scattering vector *q* of the three microgels. Solid lines donate the fitted curves with a fuzzy-sphere model (DC) or a sphere model (DS and HOMO).

For simplicity, we adopt osmotic deswelling as a criterion to clarify the softness of the synthesized core–shell microgels. The ability of a microgel to swell or deswell is directly linked to its deformability and, by extension, its softness. As proposed by Scotti et al.,^4^ the ratio between the microgel radius at infinite dilution, *R*(*c* → 0), and its radius in a reference state above the volume phase transition temperature (VPTT), *R*_ref_, serves as a quantitative descriptor of deswelling

1 $$S_{D}=\frac{R\left(c\to 0\right)}{R_{ref}}$$

A comparable *S*_D_ is obtained for HOMO (*S*_D_ = 2.277) and DS (*S*_D_ = 2.145) microgels, estimated by hydration diameters at 20 and 40 °C via dynamic light scattering (DLS) (Figure 1c). These values are significantly higher than that of DC microgels (*S*_D_ = 1.865), indicating greater softness. In this context, a higher *S*_D_ corresponds to increased microgel deformability. Within the framework of the Flory–Rehner theory,^34–36^ the total free energy (Δ*F*) of polymeric networks comprises mixing (Δ*F*_mix_) and elastic (Δ*F*_el_) contributions, which can be linked to *S*_D_

2 $$\frac{\Delta F\left(S_{D}\right)}{k_{B}T}=N_{m}\left[\left({S_{D}}^{3}-1\right)\ln\left(1-\frac{1}{{S_{D}}^{3}}\right)+\chi \left(1-\frac{1}{{S_{D}}^{3}}\right)\right]+\frac{3}{2}N_{c}\left({S_{D}}^{2}-\ln S_{D}-1\right)$$

where *k*_B_ (or *k*) is the Boltzmann constant, *T* is the absolute temperature, *N*_m_ and *N*_c_ are the number of monomer and chains in the polymer networks, and χ is the Flory solvency parameter. Assuming χ = 0.45 in the collapsed state (*T* = 313 K, *R*_h_ ∼ 50 nm),^34^ the mixing free energy yields a nearly identical level across architectures, i.e., Δ*F*_mix,DC_ = −0.538*N*_m_ *k*_B_ *T*, Δ*F*_mix,DS_ = −0.543*N*_m_ *k*_B_ *T*, and Δ*F*_mix,HOMO_ = −0.545*N*_m_ *k*_B_ *T*. Even under a thorough profiling of χ from 0 to 1, Δ*F*_mix_ remains essentially unchanged between the three (Figure S2). In contrast, elastic contribution differs markedly, i.e., Δ*F*_el,DC_ = 2.78*N*_c_ *k*_B_ *T*, Δ*F*_el,DS_ = 4.26*N*_c_ *k*_B_ *T*, and Δ*F*_el,HOMO_ = 5.04*N*_c_ *k*_B_ *T*. Thus, internal cross-linking architecture primarily governs elastic free energy, while mixing contribution is dominated by polymer–solvent interactions.

Building on above observations, the osmotic pressure (Π_os_) follows the order DC < DS < HOMO, given by the relation of Π_os_ and Δ*F* ^34^

3 $$\Pi_{os}=-\frac{N_{A}}{v_{s}}\frac{\partial \Delta F}{\partial N_{s}}$$

where *v*_s_ is the molar volume of the solvent, *N*_s_ is the number of solvent molecules in the microgel, and *N*_A_ is the Avogadro’s constant. In general, osmotic pressure within microgels is governed by dissociated counterions and electrostatic forces (due to fixed charges and mobile ions inside).^37,38^ Our estimation further reveals that elastic force from polymer network plays a decisive role in regulating osmotic deswelling. Under thermal agitation or high compression, interpenetration and entanglement of dangling chains amplify interfacial stress and enhance network elasticity—a phenomenon explored in the following sections.

### Radial Distribution Function Provides a Reliable Measure of Self-Assembly and Interparticle Potential

Having established the softness of individual microgels, we now focus on their collective behavior. Specifically, to probe how internal architecture influences interfacial properties, microgel monolayers were deposited at the air/water interface using the Langmuir–Blodgett technique (see Methods in the Supporting Information). For DC microgels, a hexagonal-like, nonclose-packed arrangement is observed during initial compression, persisting up to a surface pressure (Π) of approximately 27.5 mN/m (Figure 2a). Due to the low polymer density in the corona, only the core region is visible in AFM images,^39^ supporting the presence of corona–corona contacts. A slight increase in Π by ∼0.5 mN/m triggers sudden clustering in cores, accompanied by a divergence in interparticle distance (Π = 28 mN/m, Figure 2a). The apparent microgel size—likely corresponding to the core—continues to decrease, culminating in the formation of large aggregates arranged in a random close-packed configuration (e.g., Π = 30.5 mN/m, Figure 2a). These isolated clusters become interconnected via cross-bridging microgels and eventually reorganize into a compact monolayer, exhibiting recrystallization into hexagonal close-packed lattices with multiple orientations (Π = 32.5 mN/m, Figure 2a). This behavior mirrors the classical crystal–liquid–colloidal gel transition observed in regular PNIPAM microgels under thermal stimuli in bulk or near-wall confinement.^40,41^ In contrast, microgels with dense-shell (DS) or homogeneously cross-linked (HOMO) architectures exhibit a smooth variation in interparticle distance across the entire compression range, even in ultracondensed regimes. These microgels remain randomly distributed, forming amorphous assemblies at the interface without cluster formation (Figure 2b,c). For HOMO microgels—characterized by the most loosely cross-linked networks—the originally spherical particles undergo faceting, adopting polygonal geometries.^42^ The boundaries between neighboring microgels become indistinct, indicating a high degree of percolation across a broad pressure range (5 mN/m < Π < 30 mN/m, Figure 2c). It is worth noting that clustering of microgels has recently been linked to capillary effect during deposition and drying, as revealed by combined in situ and ex-situ observations.^16,43–48^ In our study, however, capillarity-driven (or compression-induced) clustering was only observed for DC microgels, but not for DS or HOMO. This finding highlights the role of interchain entanglements and percolation in suppressing microgel clustering during dewetting on glass substrates.

**Figure 2:**
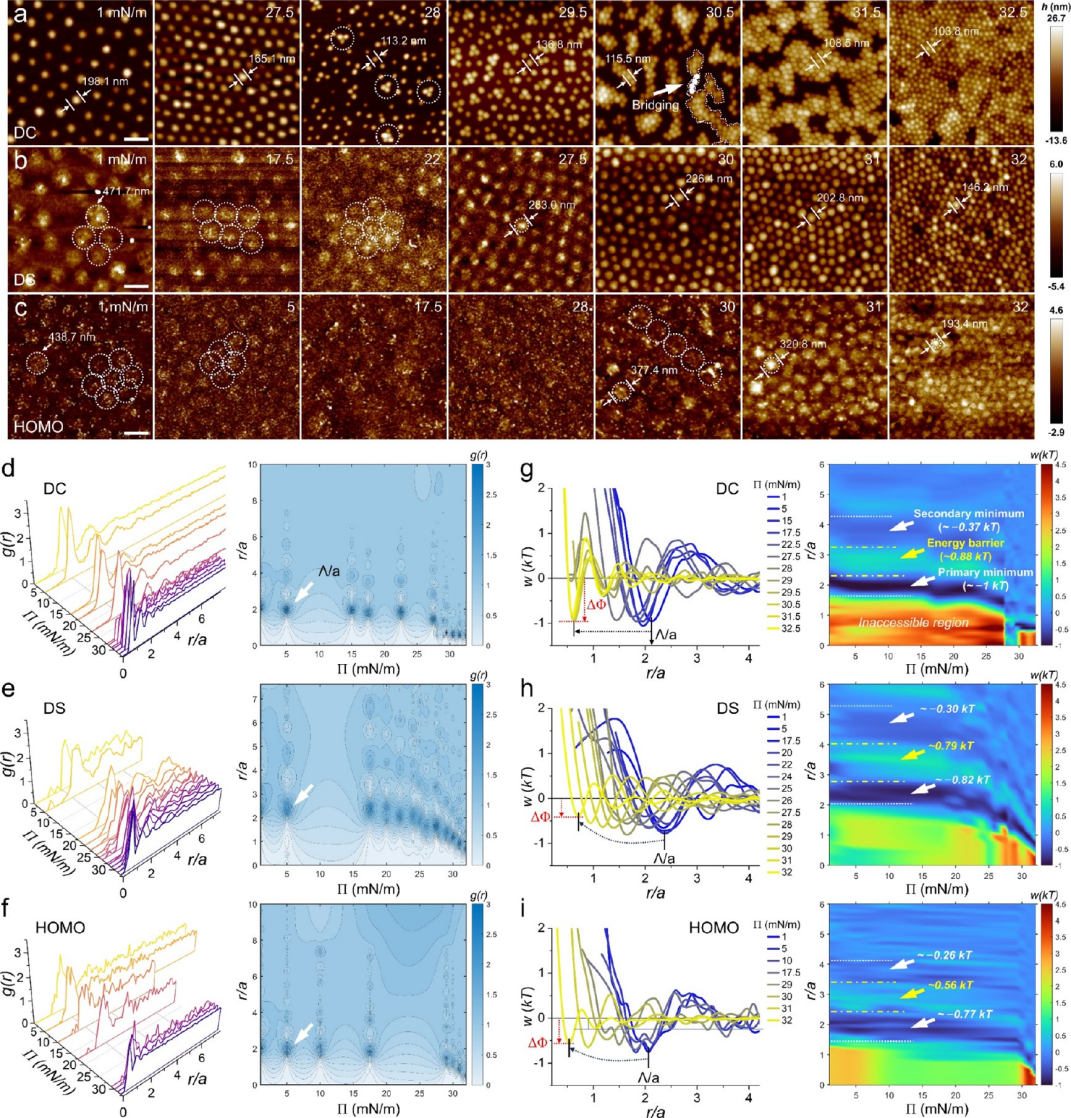
AFM images taken under dry conditions after deposition of the DC (a), DS (b) and HOMO (c) microgels onto precleaned glasses. Dotted circles indicate the emergence of clustering. Arrow indicates the bridging effect for the isolated clusters. Color bars donate the value of the height. Scale Bar: 500 nm. Waterfall and two-dimensional (2D) plots of the radial distribution function, *g*(*r*), for the DC (d), DS (e) and HOMO (f) microgels as a function of the surface pressure Π. Arrows indicate the locations of the first peak in *g*(*r*), i.e., the nearest neighbor distance, Λ. Plots of the interparticle potential profiles *w*(*r*) for the DC (g), DS (h) and HOMO (i) microgels as a function of Π. Vertical dotted arrows indicate the attractive potentials at the Λ. Right: simulations of the corresponding interparticle potentials that mark the primary minima ΔΦ, secondary minima ΔΦ_second_, energy barriers between ΔΦ and ΔΦ_second_, and the uncompressible regions at short distances. Color bars represent the value of *w*(*r*) in *kT*.

To elucidate the ordering and interparticle separation of microgels at the air/water interface, a radial distribution function, *g*(*r*), is employed to quantify structural parameters such as the nearest neighbor distance, Λ (Figures 2d–f and S3), as previously described.^29^ Here, Λ is normalized by the hydration diameter *a* to facilitate comparison of the relative compressibility among the three microgels. Waterfall and 2D plots of *g*(*r*) reveal an abrupt decrease in Λ at a surface pressure of Π = 28 mN/m for DC microgels, indicating rapid nucleation and clustering of the dense cores. In contrast, DS and HOMO microgels exhibit a gradual decrease in Λ, suggesting steady intercorona interactions under compression. Interparticle interactions are further estimated via Boltzmann inversion of *g*(*r*), i.e., *w*(*r*) = −*kT* ln­[*g*(*r*)], where *w*(*r*) represents the interparticle potential of mean force—an approximation of the pairwise interaction potential in the limit of infinite dilution.^49,50^ Under this framework, the deepest minima ΔΦ in the potential energy profiles are located at the first peak of *g*(*r*) (Figure 2g–i, left panel and Figure S4). Simulation of the energy landscape, plotted as a function of surface pressure and interparticle distance, reveal the strength and range of interparticle forces within the monolayer (Figure 2g–i, right panel). At low surface pressures, DC microgels exhibit a primary minima ΔΦ ∼ −1 *kT*, indicating that the fuzzy shell contributes to the interchain attraction between neighboring particles. In contrast, DS and HOMO microgels display a relatively weaker ΔΦ, along with weaker secondary minima (ΔΦ_second_) and lower energy barriers between the two. Additionally, a pronounced repulsive energy barrier (∼2–4 *kT*) is observed at short interparticle distances (corresponding to high compressions). This high barrier reflects an incompressible regime at the interface, where loosely cross-linked polymer networks can be compressed into shorter separations without undergoing clustering or isostructural phase transition.

### Thermal Deswelling and Interfacial Elasticity

Microgels undergo swelling/deswelling under thermal agitation or deform under high compression, thereby tuning their core–corona structure and interchain entanglements within confined monolayers. The preceding sections have established correlations between single-particle architecture, softness, and collective assembly at interfaces. Importantly, the radial distribution function *g*(*r*) provides a direct link between surface pressure Π and nearest neighbor distance Λ, thus enabling a possibility to evaluate monolayer elasticity from these two parameters.

We first estimate the effect of temperature on monolayer compressibility by monitoring Π under steady compression across the VPTT using a Langmuir trough. The surface area *A* was normalized by the mass of microgels to generate compression isotherms (Figure 3a). For DC microgels, the Π–*A* curves measured at three different temperatures spanning the VPTT initially overlap during early compression, but diverge into distinct trajectories as Π approaches ∼25 mM/m (inset, Figure 3a, left panel). In contrast, the compression isotherms for DS and HOMO microgels display the opposite trend: they diverge at low surface pressures but converge into a single curve under high compression (inset, Figure 3a, middle and right panels). Assuming that the occupied area by individual microgels at the air/water interface remains temperature-independent at fixed Π—as previously reported^22^ and directly evidenced by AFM images (Figure 3b–d)—these divergent overlapping behaviors in the compression isotherms reflect the heterogeneous distribution of softness within core–shell microgels, particularly in the outer corona region.

**Figure 3:**
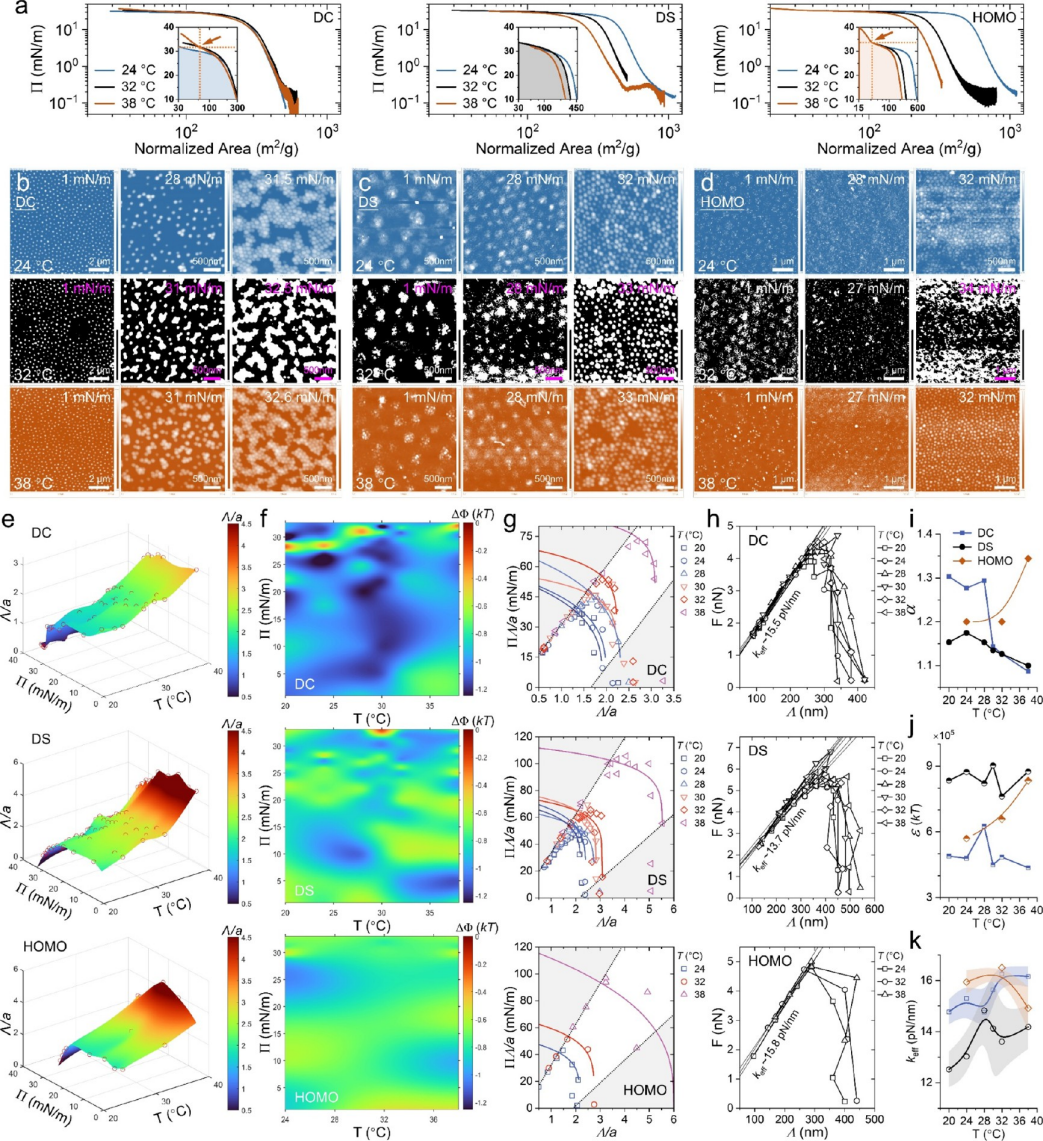
(a) Compression isotherms across the VPTT for the DC, DS and HOMO microgels. (b–d) show the self-assembly of the DC (b), DS (c), and HOMO (d) microgels under different surface pressures Π. (e) 3D plots of the nearest neighbor distance Λ*/a* as functions of Π and temperature. (f) 2D plots of the interparticle attractive potential ΔΦ at corresponding Λ/*a* as functions of Π and temperature. (g) Plots of the modified surface pressure Π-versus-Λ/*a*. Solid curves denote the best fit of the generalized Hertzian model, described by eq 5. (h) Plots of the interparticle force *F*-versus-Λ. Solid lines denote the linear relations, where the elastic coefficient *k*_eff_ is deduced from the slopes. (i–k) show the temperature dependences of the power-law exponent α (i), the energy scale (j), and the *k*_eff_. Shadows denote the error bars.

Using *g*(*r*) and Boltzmann inversion, we next quantify the Λ and the primary minima ΔΦ—a measure of the attractive interaction between neighboring microgels—via both 2D and 3D plots (see Figures 3e and S5 for Λ, Figure 3f for ΔΦ). Notably, at low temperatures below the VPTT, all three types of microgels exhibit a universal uncompressed interparticle spacing (Λ/*a* ∼0.5), indicating a common baseline for interparticle separation that is independent of internal architecture and reflects the volume exclusion effect of polymer chains. However, Λ/*a* exhibits a pronounced temperature dependence. At temperatures above the VPTT, e.g., *T* = 38 °C, the minimum Λ/*a* increases to ∼0.8 for DC microgels, ∼1.5 for DS microgels, and ∼2 for HOMO microgels (Figure S6), reflecting their distinct thermally induced deswelling behaviors under 2D confinement. Concurrently, the interaction potential ΔΦ also shows distinct temperature and pressure dependence. DC microgels show a more negative average ΔΦ of approximately −1.2 *kT*, distributed inhomogeneously across the 2D landscape of Π and temperature (Figure 3f). In contrast, DS microgels display intermediate values (−0.6 *kT* to −1.0 *kT*), while HOMO microgels maintain a relatively weak attraction (∼−0.6 *kT*). The most pronounced minima in ΔΦ for DC microgels occur near *T* ∼30 °C, close to the VPTT, indicating maximal interparticle attraction driven by optimal chain flexibility and interfacial confinement.

Extracting the key structural parameter Λ/*a* from the *g*(*r*) at various Π enables us to further evaluate the monolayer elasticity. To accommodate the deformability of soft microgels, we adopt a generalized Hertzian potential with a relaxed power-law exponent, expressed as^39,51,52^

4 $$U\left(\Lambda \right)=\frac{\varepsilon }{\alpha }{\left(1-\frac{\Lambda }{a}\right)}^{\alpha }\Theta \left(1-\frac{\Lambda }{a}\right)$$

where ε is the energy scale, Θ is the Heaviside step function, and α is the power-law exponent that defines the shape of the potential. This model captures the soft repulsive interactions allowing overlaps between microgels in a semidilute regime. Given a hexagon-like, nonclose-packed arrangement, the surface pressure Π can be derived as^39^

5 $$\Pi =-\frac{\partial E_{hex}}{\partial A_{hex}}=\frac{\varepsilon \sqrt{3}}{a\Lambda }{\left(1-\frac{\Lambda }{a}\right)}^{\alpha -1}\Theta \left(1-\frac{\Lambda }{a}\right)$$

where *E*_hex_ is the potential energy of a unit cell in a two-dimensional hexagonally packed arrangement, given by *E*_hex_ = 3*U*(Λ), and $A_{hex}=\Lambda^{2}\sqrt{3}/2$ . Equation 5 provides a robust description of the surface pressure Π as a function of the normalized interparticle distance Λ/*a* for all three types of microgels prior to clustering (i.e., in the regime of large Λ/*a*, involving corona–corona contacts), as shown by the solid curves in Figure 3g. The best-fit values of the power-law exponent α range from approximately 1.1 to 1.35 (Figure 3i), indicating a significant deviation from the ideal harmonic potential (α = 2) and the classical Hertzian repulsion model (α = 2.5 in three dimensions).^53^ Such a reduced exponent reflects that the repulsion force between approaching microgels is more sensitive to small compressions than to extensive overlaps, highlighting the nonlinear nature of their contact mechanics.^39^ In addition, temperature-dependent variations in α reveal architecture-specific behaviors. DC microgels exhibit α ≈ 1.3 at low temperatures, but it rapidly drops to ∼1.1 across the VPTT, signifying a shift from soft repulsion to hard-sphere-like interactions due to pronounced deswelling of the outer layer. DS microgels maintain the lowest α, reflecting their inherently stiff corona. Conversely, HOMO microgels display an increase in α from ∼1.2 to ∼1.35 (approaching 1.5), consistent with ultrasoft-potential fluid simulations of highly deformable particles.^51^ In addition, the ensembled interaction energy scale ε could be found in Figure 3j. Notably, for small Λ/*a*, a linear relation better describes the surface pressure behavior than the Hertzian model, capturing the rapid decrease in Λ at high Π as the system approaches jamming. Motivated by this observation, the interparticle force can be expressed as

6 $$F\left(\Lambda \right)=-\frac{\partial U}{\partial \Lambda }=-\frac{1}{3}\frac{\partial E_{hex}}{\partial A_{hex}}\frac{\partial A_{hex}}{\partial \Lambda }=\frac{\varepsilon }{a}{\left(1-\frac{\Lambda }{a}\right)}^{\alpha -1}\Theta \left(1-\frac{\Lambda }{a}\right)$$

Plotting the interparticle force *F*(Λ) against the nearest neighbor distance Λ reveals a remarkable superposition: all force measurements across different temperatures collapse onto a single master curve (Figure 3h). A linear dependence emerges for Λ < ∼300 nm, delineating the boundary of the linear viscoelastic (LVE) regime for condensed soft particles confined in two dimensions. To assess the influence of internal architecture on interfacial elasticity, we present the elastic coefficients, *k*_eff_, in Figure 3k. Similar to the scaling exponent α, *k*_eff_ exhibits architecture- and temperature-dependent variations. For DC microgels, a step-like increase in *k*_eff_ is observed across the VPTT, reflecting enhanced core–core repulsion under thermal agitation. DS microgels display the lowest elastic responses throughout the temperature ramp, likely due to low cross-linked core regions (resembling cavities in hollow microgels) that dissipate energy via shrinkage or bending near the jamming regime.^54,55^ In contrast, HOMO microgels exhibit the highest elasticity among the three architectures, but *k*_eff_ decreases above the VPTT, suggesting softening or melting of the interfacial thin layer. Strikingly, our results indicate that enhanced interfacial elasticity arises from microgels with more homogeneously or loosely cross-linked—i.e., “softer”—polymeric networks, rather than from those with dense cores or shells. This finding resonates with earlier observations that softer microgels promote stronger elastic free energy (Δ*F*_el_) and osmotic deswelling (Π_os)_. It suggests that heterogeneity in internal structure plays a more pivotal role in interfacial mechanics than previously anticipated. Given the complete flattening of homogeneously cross-linked networks at the interface, we speculate that the enhanced elasticity originates from a greater number of polymer chains and cross-linking junctions actively participating in the transport of interfacial stress. This mechanism highlights the critical interplay between microgel architecture and emergent mechanical properties under 2D confinement.

### Salt Effects—Interchain Electrostatic Interactions

Having extracted the interfacial elasticity of microgel monolayers, we now move to investigating how interchain electrostatic interactions in the liquid phase influence monolayer mechanics. To this end, we systematically analyzed compression isotherms across a gradient of ionic strength, i.e., varying sodium chloride concentrations *c*(NaCl). These isotherms were normalized into master curves using anchor points defined by the apparent surface pressure Π_ap_ and area *A*_ap_, as shown in Figures 4a and S7. A clear superposition is achieved for loosely cross-linked polymeric networks—specifically, DS and HOMO microgels—indicating a consistent elastic response across varying salt concentrations. In contrast, the compression curves for DC microgels fail to collapse in the high-Π and high-*A* regions (Figure 4a, top panel), suggesting that ionic strength exerts a more pronounced influence on the compressibility and softness of core–shell microgels with regular cross-linking. The *A*_ap_ for DS and HOMO microgels spans a wide range, from approximately 300 m^2^/g in pure water to nearly 900 m^2^/g at high *c*(NaCl) (Figure 4b, solid lines). This substantial variation highlights the high compressibility of loosely cross-linked networks at the air/water interface. Additionally, the Π_ap_ tends to increase slightly upon salt addition compared to pure water, although its dependence on *c*(NaCl) remains nonmonotonic and temperature-sensitive.

**Figure 4:**
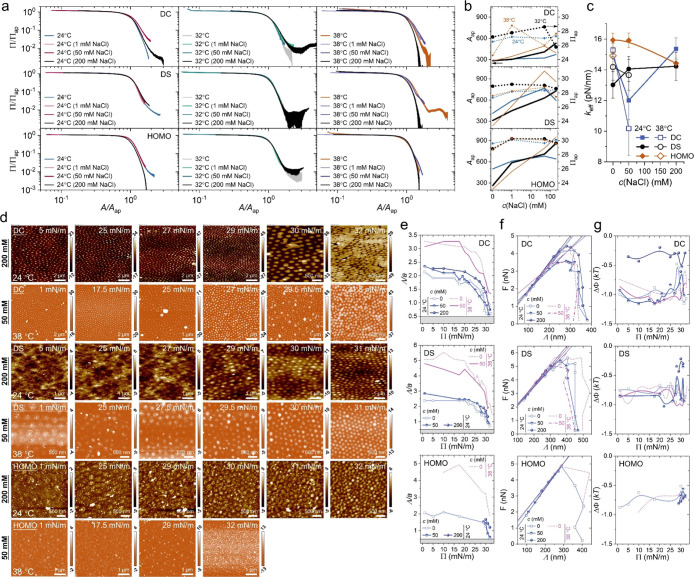
(a) Normalized compression isotherms across the VPTT for the DC, DS and HOMO microgels under different concentrations of NaCl. (b) The anchor points for the surface pressure Π_ap_ (dotted lines) and compression area A_ap_ (solid lines). (c) Plots of the elastic coefficient *k*_eff_-versus-*c*(NaCl) for the DC, DS and HOMO microgels at two temperatures across the VPTT. (d) Shows the AFM images obtained at high *c*(NaCl). Color bars denote the height. (e) Plots of the interparticle distance Λ*/a*-versus-Π at several *c*(NaCl). (f) Plots of the interparticle force *F*-versus-Λ. Solid lines denote the linear relations, where the elastic coefficient *k*_eff_ in (c) is deduced. (g) Plots of the attractive potential ΔΦ-versus-Π at several *c*(NaCl).

We next turn our attention to the self-assembly behavior of microgel monolayers and analyze their structural and mechanical properties under varying ionic strengths. Figure 4d presents AFM images of microgel monolayers formed at high *c*(NaCl), acquired at both low (24 °C) and high (38 °C) temperatures across the VPTT. These conditions allow us to evaluate the role of salt in modulating interparticle interactions in both swollen and deswollen states. Notably, no clustering is observed for DC microgels under high ionic strength, even at temperatures well above the VPTT. Previous studies have shown that microgels can undergo a two-dimensional glass-to-colloidal gel phase transition across the VPTT at high packing densities.^16^ Hence, the addition of salt appears to weaken interparticle interactions within the monolayer, thereby altering the 2D phase behavior. This is supported by a measurable decrease in the attractive potential energy ΔΦ between neighboring microgels at high *c*(NaCl) (e.g., 200 mM), as shown in Figure 4g. The corresponding Λ/*a* for assembled structures under high *c*(NaCl) conditions is presented in Figure 4e. Interestingly, we observe an increase in Λ/*a* with increasing *c*(NaCl) below the VPTT, and a decrease above the VPTT. While increased ionic strength is typically expected to screen electrostatic repulsion and reduce interparticle separation—potentially promoting clustering or sedimentation—this behavior does not manifest in the two-dimensional interfacial system studied here. These findings suggest that aqueous solutions with high salt concentrations, traditionally considered poor solvents for microgels in bulk,^56^ may instead act as “good” solvents at the interface, enhancing microgel spreading and suppressing aggregation. A more detailed investigation would be presented in next sections.

Using eq 6, we extract the linear relationship between interparticle force and separation distance, enabling quantification of the interfacial elasticity under varying ionic strengths (Figure 4c,f). Remarkably, the aforementioned anomalous behavior—where softer microgels can form more robust polymeric thin layers—persists at intermediate salt concentrations (i.e., 50 mM). Upon further increasing the salt concentration, the interfacial elasticity exhibits distinct trends depending on the microgel architecture: it slightly decreases for HOMO microgels, remains largely unchanged for DS microgels, and increases significantly for DC microgels. This complex behavior underscores the architecture-dependent role of ionic strength in modulating interfacial mechanics. For HOMO and DS microgels, flattening of loosely or uniformly cross-linked networks at the interface minimizes the impact of salt-induced screening. In contrast, DC microgels, with their pronounced core–shell structure and swollen dangling chains, respond more sensitively to ionic strength, leading to the disappearance of clustering and more complex interfacial elasticity under salt effects.

### Co-nonsolvency—Polymer–Solvent Interactions

So far, we have examined the elasticity of microgel monolayers in pure aqueous environments. We further proposed that bulk-poor solvents, such as high-salt aqueous solutions, may transition to good solvents at interfaces for PNIPAM microgels. To investigate how solvent quality modulates assembly structure and monolayer mechanics at fluid interfaces, ethanol (EtOH) was introduced as a cosolvent in water. The EtOH–water mixture is known to act as a poor solvent for PNIPAM single chains and cross-linked microgels. Specifically, PNIPAM chains are soluble in pure EtOH at all temperatures and in water below the VPTT, but become insoluble in EtOH–water mixtures—a phenomenon referred to as cononsolvency.^57,58^ While this behavior has been extensively studied in bulk systems, its implications for interfacial monolayers remain largely unexplored. To address this gap, we first examined the compression behavior of microgel monolayers formed at varying EtOH: water volume ratios. As shown in Figure 5a, the compression isotherms exhibit a pronounced shift toward lower values of both surface pressure Π and area *A*, particularly at higher EtOH content. A reduction in Π is also evident during the initial stages of compression. These observations suggest that EtOH molecules significantly suppress polymer chain extension and hydration, thereby weakening interparticle interactions at the air/water interface. A decrease of the hydration diameter and the zeta potential of microgels dispersed in EtOH–water mixtures could be found in Figure S8.

**Figure 5:**
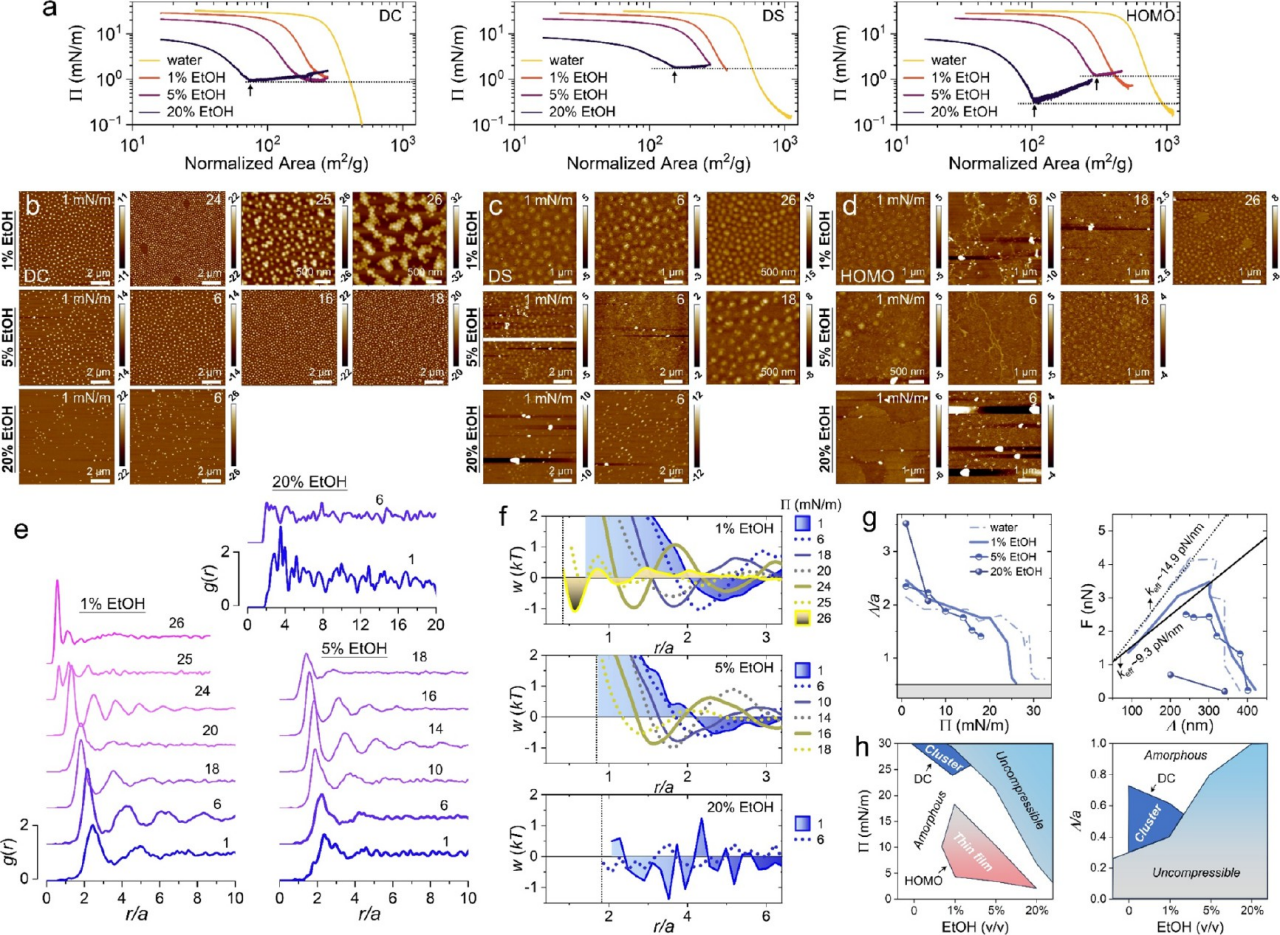
(a) Compression isotherms obtained at various EtOH–water mixtures. (b–d) AFM images obtained at different volume ratios of EtOH for the DC (b), DS (c), and HOMO (d) microgels. Color bars denote the height. (e) Radial distribution function *g*(*r*) obtained at different volume ratios of EtOH for the DC microgels. Numbers on top of the *g*(*r*) denote the surface pressure Π. (f) Plots of the interparticle potential profiles *w*(*r*) for the corresponding *g*(*r*) in (e). (g) Left: plots of the interparticle distance Λ/*a*-versus-Π at different volume ratios of EtOH. Right: plots of the interparticle force *F*-versus-Λ. Solid lines indicate the elastic coefficient *k*_eff_. (h) Plots of the 2D phase diagrams for the microgel monolayers as functions of the surface pressure Π (left), the interparticle distance Λ/*a* (right) and the volume ratios of EtOH.

We next investigate the interfacial self-assembly behavior of microgel monolayers upon introducing ethanol (EtOH) into the bulk aqueous phase. AFM images (Figure 5b–d) reveal that at 20 vol % EtOH, only a limited number of microgels can adsorb at the interface. For loosely cross-linked polymer networks at low Π (e.g., Π = 6 mN/m for DS microgels at 5 vol % EtOH in Figure 5c; Π = 6 mN/m for HOMO microgels at 1 vol % and 5 vol % EtOH in Figure 5d), the location of microgels becomes indistinguishable. Instead, a continuous polymeric thin film with thickness on the nanometer scale is observed, indicating a transition from discrete particle adsorption to film-like spreading. To further probe interparticle interactions and mechanical properties, we focus on DC microgel monolayers while varying the EtOH volume fraction. The corresponding *g*(*r*) and *w*(Λ) are shown in Figure 5e,f. Compared to pure water, the normalized interparticle distance Λ/*a* shifts toward lower surface pressure in EtOH–water mixtures (Figure 5g, left panel), suggesting that the cosolvent increases microgel separation at the interface. The force–distance relationship (Figure 5g, right panel) reveals a reduced effective interfacial elasticity (*k*_eff_ ∼9.3 mN/m) under cononsolvency conditions. Integrating above observations leads to a phase diagram for the microgel monolayers in two dimensions (Figure 5h). Regular cross-linked microgel clusters are only stable at extremely low EtOH content, whereas homogeneously cross-linked networks can form thin films within a defined range of surface pressure. A broad incompressible regime emerges in EtOH–water mixtures, confirming that cononsolvency effects persist and significantly influence interfacial behavior of microgels in 2D systems.

Until now, we have examined the compressibility and elasticity of microgel monolayers under varied aqueous conditions, including changes in salt concentration and cononsolvency. The surface pressure–area (Π–*A*) compression curves evoke parallels with classical scaling theories developed for linear polymer chains. In the semidilute regime, the relationship between surface pressure and surface coverage can be described by^19^

7 $$\Pi \propto \Gamma^{m_{2D}}$$

where Γ ∼*A* ^–1^ is the surface coverage, and the scaling exponent *m*_2D_ is related to the solvent quality parameter υ by

8 $$m_{2D}=2\upsilon /\left(2\upsilon -1\right)$$

The scaling theories have predicted that the value of υ_2D_^+^ = 3/4 for a good solvent, υ_2D_^θ^ = 4/7 for a theta solvent, and υ_2D_^–^ = 1/2 for a poor solvent for the space-filling 2D polymers.^59–61^ For the microgel monolayers studied here in the 2D systems, we reanalyze the Π–*A* curves obtained at varied *c*(NaCl) (Figure 4a) and in EtOH–water mixtures (Figure 5a). In the semidilute regimes, the slopes in the double-logarithmic Π–*A* isotherms yield a value of υ ∼0.57, regardless of salt concentration or temperature (Figure 6a). This scaling behavior suggests that NaCl solutions—although poor solvents in bulk—act as theta solvents for PNIPAM networks at the air/water interface. According to Flory–Huggins theory, theta solvent conditions imply ideal polymer behavior, where chains adopt random-walk conformations and exhibit no energetic preference for mixing or adsorption.^62^ In the condensed regimes, the scaling exponent *m*_2D_^con^, shown in Figure 6b, reveals a stronger correlation between surface pressure and interparticle distance for DC microgels compared to DS or HOMO microgels, particularly at temperatures near the VPTT. For EtOH–water mixtures, fitting the Π–*A* curves to scaling theory yields υ values ranging from ∼0.56 (close to υ_2D_^θ^) to values exceeding 1, indicating that the mixed solvent behaves between a good and theta solvent for PNIPAM chains at the interface. These findings highlight the nuanced role of solvent quality in governing interfacial mechanics and self-assembly. Future experimental and theoretical studies are needed to further validate scaling-law predictions in two-dimensional soft matter systems.

**Figure 6:**
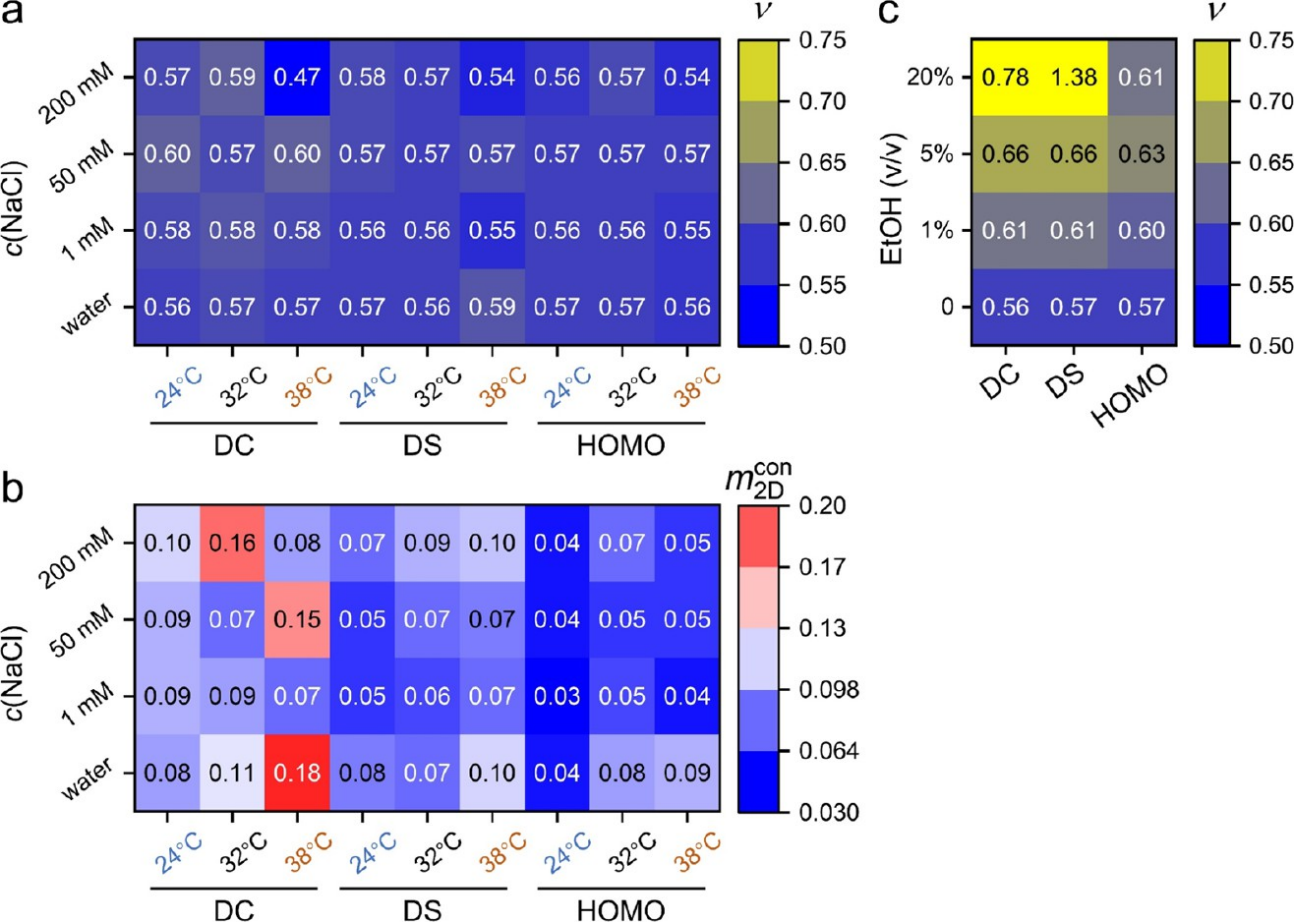
(a) Solvent quality υ obtained from the double-logarithmic Π–*A* isotherms as functions of the *c*(NaCl) and temperature. Colors indicate the values of υ. (b) Scaling exponent in the condensed regimes, *m*_2D_^con^, as functions of the *c*(NaCl) and temperature. Colors indicate the values of *m*_2D_^con^. (c) Solvent quality υ obtained at different volume ratios of EtOH. Colors indicate the values of υ.

## Conclusions and Discussion

In this study, we investigated the intermacromolecular interactions and interfacial elasticity of microgel monolayers composed of core–shell microgels with varied internal architectures. Guided by osmotic deswelling and Flory–Rehner theory, we found that loosely or homogeneously cross-linked polymer networks exhibit pronounced softness, accompanied by enhanced intrachain repulsion within individual microgels. The interparticle potential energy was further quantified via Boltzmann inversion of the radial distribution function *g*(*r*). Notably, regularly cross-linked microgels displayed a more negative primary minimum (ΔΦ ≈ −1 *kT*) in the two-dimensional energy landscape at low surface pressure Π, suggesting that the fuzzy shell architecture promotes interchain attraction among nearest neighbors.

To assess interfacial elasticity, we applied a generalized Hertzian model in the semidilute regime. The extracted power-law exponent (α ≤ 1.35) indicates significant chain entanglement under interfacial confinement. In contrast, the ultracondensed regime revealed a linear dependence between interparticle force and nearest neighbor distance Λ, enabling the determination of the effective interfacial elastic modulus, *k*_eff_. Surprisingly, the softer microgels—those with loosely or homogeneously cross-linked networks—exhibited higher interfacial elasticity (∼16 pN/nm), underscoring the critical role of internal architecture in governing 2D mechanical behavior.

We further explored the influence of salt concentration and solvent quality on monolayer elasticity. While ionic strength modulated elasticity in architecture-dependent ways, EtOH–water mixtures (poor solvents for PNIPAM in bulk) significantly reduced interfacial elasticity (e.g., *k*_eff_ < 10 mN/m at 1 vol % EtOH), confirming the presence of cononsolvency effects in two dimensions. Interestingly, scaling analyses of compression isotherms suggest that poor solvents such as NaCl solutions or EtOH–water mixtures may act as theta or even good solvents for PNIPAM microgels at the air/water interface.

Overall, these findings highlight the profound impact of internal microgel architecture on collective interfacial mechanics, offering new insights into the design of tunable soft polymeric materials in two-dimensional systems.

## Supplementary Material



## Notes

Experimental methods, materials, and microgel characterization (PDF)
