# Supplementary material for: Effect of Internal Architecture on the Elasticity of Microgel Monolayers at the Air/Water Interface
Source: Macromolecules. 2025 Dec 10;58(24):13191–203. doi: 10.1021/acs.macromol.5c02434 (PMC12752682; doi:10.1021/acs.macromol.5c02434)
Supplement: Supplementary file 1 [file ma5c02434_si_001.pdf]

Supporting Information

Effect of Internal Architecture on the Elasticity of Microgel Monolayers at the Air/Water Interface

Wei Liu,[†,#] Li Zhang,[†,#] Zehua Han,[‡] He Cheng,[*,‡] Hailin Li,[ǁ] Xiangjun Gong,[ǁ] Hang Jiang,[†] Yuwei Zhu,[*,†] and To Ngai[*,§]

[†]The Key Laboratory of Synthetic and Biological Colloids, Ministry of Education & School of Chemical and Material Engineering, Jiangnan University, Wuxi 214122, China

[‡]Spallation Neutron Source Science Center (SNSSC), Dongguan 523803, China

[ǁ]Faculty of Materials Science and Engineering, South China University of Technology, Guangzhou 510640, China

[§]Department of Chemistry, The Chinese University of Hong Kong, Shatin, N.T., Hong Kong 999077, China

Corresponding author

Dr. He Cheng, email: chenghe@ihep.ac.cn
Spallation Neutron Source Science Center (SNSSC), Dongguan 523803, China

Dr. Yuwei Zhu, email: uvyzhu@gmail.com
The Key Laboratory of Synthetic and Biological Colloids, Ministry of Education & School of Chemical and Material Engineering, Jiangnan University, Wuxi 214122, China

Dr. To Ngai, email: tongai@cuhk.edu.hk
Department of Chemistry, The Chinese University of Hong Kong, Shatin, N.T., Hong Kong 999077, China

Materials

N-isopropylacrylamide (NIPAM, >98%, Tokyo Chemical Industry, Japan), *N,N'*-methylenebisacrylamide (BIS, 99%, Shanghai Aladdin Biochemical Technology, China), Potassium persulfate (KPS, AR, Sinopharm Chemical Reagent, China), Sodium dodecyl sulfate (SDS, ≥99%, Sinopharm Chemical Reagent, China), and Sodium chloride (NaCl, AR, Sinopharm Chemical Reagent, China) were used for synthesis as received. Ethanol (EtOH, AR, Sinopharm Chemical Reagent, China) and Deionized water (18.2 MΩ·cm, Smart-S15, HHitech, China) were utilized to prepare bulk solutions for Langmuir tough.

Microgel Synthesis

The synthesis for regular cross-linked microgels (*i.e.*, dense-core (DC) microgels) was done by a standard precipitation polymerization method with surfactant and was already described in detail elsewhere.[1,2] In brief, a mixture of NIPAM (17.461 g), BIS (1.217 g), and SDS (0.224 g) was dissolved in 592 mL D.I. water and put into a three-neck round-bottom flask equipped with a magnetic stirrer, a reflux condenser, and a nitrogen gas inlet. After stirring for 30 min at 70 ℃ under nitrogen purge, KPS (0.487 g) dissolved in 8 mL D.I. water was injected into the flask to initiate the polymerization. The reaction was maintained at 70 ℃ for 4 hours to achieve the DC microgels.

The synthesis for dense-shell (DS) microgels and homogeneously (HOMO) cross-linked microgels was similar to that for the DC microgels, where the timing of cross-linker introduction during polymerization were well controlled, see Figure 1a for detailed protocol. Briefly, for DS microgels, 50% monomer and 100% crosslinker were introduced into the polymerization after initiation for 1 hour. For HOMO microgels, 100% crosslinker was injected continuously into the flask at a speed of 60 mL/h for 1 hour. For all microgels, to remove unreacted chemicals and surfactants, the dispersion was purified via filtration and dialysis for 7 days with daily twice replacement of D.I. water, and concentrated using a rotary evaporator (HR-21M, Shanghai Huxi Industry, China).

Microgel Characterization

The hydrodynamic diameter D_h of the microgels (c = 5 × 10^{-4} g/mL) was determined by dynamic light scattering (ZetaPALS and NanoBrook Omni, Brookhaven Instruments Corporation, USA) at varied temperatures (20 to 40 °C), NaCl concentrations (1 to 200 mM), and volume ratio of EtOH (1 to 20 %).

The temperature and vol % EtOH dependences of the zeta potential ζ for the microgels were determined *via* a Zetasizer Nano (ZEN3700, Malvern Instruments Inc., U.K.) at c = 1 × 10^{-4} g/mL. The suspension was allowed to equilibrate at least for 5 minutes at each condition before measurement, and all the test was conducted for three times.

Small angle neutron scattering (SANS) experiments were performed using the Very Small Angle Neutron Scattering (VSANS) instrument BL-14 at the China Spallation Neutron Source

(CSNS).[3,4] Neutron data were collected with a wavelength range from 6 Å to 10.5 Å, employing a collimation length of 12.75 m. The minimum q can reach 0.002 $Å^{-1}$. Samples were encased in quartz cells with an optical path length of 1 mm. Data reduction was performed using the direct beam method which included measurement of the direct beam, solid angle and transmission correction as well as solvent scattering background subtraction.

To observe the assembly structure of microgels, monolayers were transferred to silicon wafers under certain surface pressures and allowed to dry in air at room temperature. The array structures of the microgels were further observed using AFM (Veeco Multimode 8, Bruker Daltonics Inc., USA and MFP-3D, Oxford Instruments, UK) in Scanasyst-Air mode, using a silicon tip on nitride lever with a spring constant of 2.8 $N\cdot m^{-1}$.

The effective volume fraction ϕ_{eff} of microgels was estimated by $\phi_{eff} = bcD_h^3/2.5$, where b is the proportionality constant between $[\eta]$ and D_h^3 based on the Huggins and Mead–Fuoss plots and $[\eta]$ is the intrinsic viscosity of the dilute microgel suspensions measured through a Ubbelohde viscometer. Microgels dispersions were prepared at various concentrations to yield two distinct ϕ_{eff} ~1 (ϕ_{eff} ~0.961 for DC, ~1.041 for DS, and ~1.110 for HOMO) and ~1.5 (ϕ_{eff} ~1.470 for DC, ~1.433 for DS, and ~1.479 for HOMO) respectively for further rheological measurements. The rheological tests were performed on a bulk rheometer (MCR302e, Anton Paar, Austria) equipped with a 25 mm plate. For oscillatory testing, frequency sweeps from 0.01 s^{-1} to 1 s^{-1} at a fixed temperature of 20 ℃ (strain: 0.5%, gap size: 0.5 mm).

Liquid-phase imaging and nanomechanical mapping were performed on a NanoWizard ULTRA Speed 2 AFM (Bruker). The specimen was mounted on the scanner stage and immersed in ultrapure water to ensure complete hydration. The probe was then lowered to the sample surface, and additional pure water was added to immerse the probe. DNP-A probe (f = 65 kHz, k = 0.350 $N\cdot m^{-1}$, Bruker) was used and the spring constant was determined before through the thermal noise method before imaging. Quantitative Imaging™ mode was employed to simultaneously record topography and mechanical properties. Images were acquired at an applied setpoint of 1.5 nN over a 3×3 μm^2 area (256 × 256 pixels) with a tip velocity of 53.6 $\mu m\cdot s^{-1}$. The vertical scan range of the z-scanner was restricted to 0.1 μm. All data were processed using JPK Data Processing software.

Langmuir Trough

Microgel monolayer at the air/water interface was prepared and compressed by a Teflon Langmuir trough (MicroTrough G2, Kibron Inc., Finland) equipped with Teflon barriers and a Wilhelmy plate. The compression isotherms were measured with a compressible area of 27999 mm^2 (inner trough dimensions: width: 80 mm, length: 405 mm, depth: 5 mm). Before measurement, the solution was spread into the Langmuir trough, and the air–solution interfacial surface pressure was then calibrated when the solution temperature reaches the set ones. After calibration, the microgel suspensions were diluted to 1×10^{-3} g/mL at desired condition, and then mixed with ethanol as a spreading agent. The interface was equilibrated for 30 minutes

before starting a compression/deposition test, thereafter the barriers were driven to compress the interface symmetrically at a constant speed of 5 mm/min.

Silicon wafers (Sinopharm Chemical Reagent, China) were cut into 10 mm × 10 mm pieces and underwent rinsing with water and ethanol, the slides were dried using nitrogen gas and subsequently cleaned for 5 minutes in an ultraviolet-ozone plasma cleaner (PDC-002, Harrick Plasma, USA) before use. To transfer the microgel monolayers formed at the air/water interface onto a solid substrate, the silicon wafer was dipped in water before spreading the microgels at the air/water interface and it was then pulled up through the microgel monolayer at the air/water interface by a small programmable dipper (LayerX (90), Kibron Inc., Finland). The barrier position was automatically adjusted to maintain the target surface pressure. The pull-up speed was 0.3 mm/min at an angle of ~40°.

Radial Distribution Function

A radial distribution function, $g_{ij}(r)$, describes the probability of a particle *j* with coordinates $\mathbf{r}_j$ locating within a thin shell of infinitesimal thickness δr at distance *r* from another particle *i* with coordinates $\mathbf{r}_i$. From the obtained AFM images, the coordinates of the microgels were firstly obtained by manual labelling of the images, then a two-dimensional radial distribution function (2D-RDF) can be computed according to

$$g^{\mathrm{2D}}(r) = \frac{1}{\rho^{\mathrm{2D}}} \left\langle \frac{1}{N} \sum_{i=1}^{N_i} \sum_{j \neq i}^{N_j} \delta(r - r_{ij}) \right\rangle$$

where $r_{ij} = |\mathbf{r}_i - \mathbf{r}_j|$, N is the total number of particles present, ρ^{2D} is the two-dimensional number density of particles, and δ is the Dirac delta function. The distances were binned with inner and outer radii of $r + n\Delta r$ and $r + (n+1)\Delta r$ with $\Delta r = 2$–4 pixels during the numerical computation of the 2D-RDFs.

To reduce the artifacts by the finite size of images, a two-dimensional periodic boundary condition was utilized as an approximation in the computation of the 2D-RDFs.

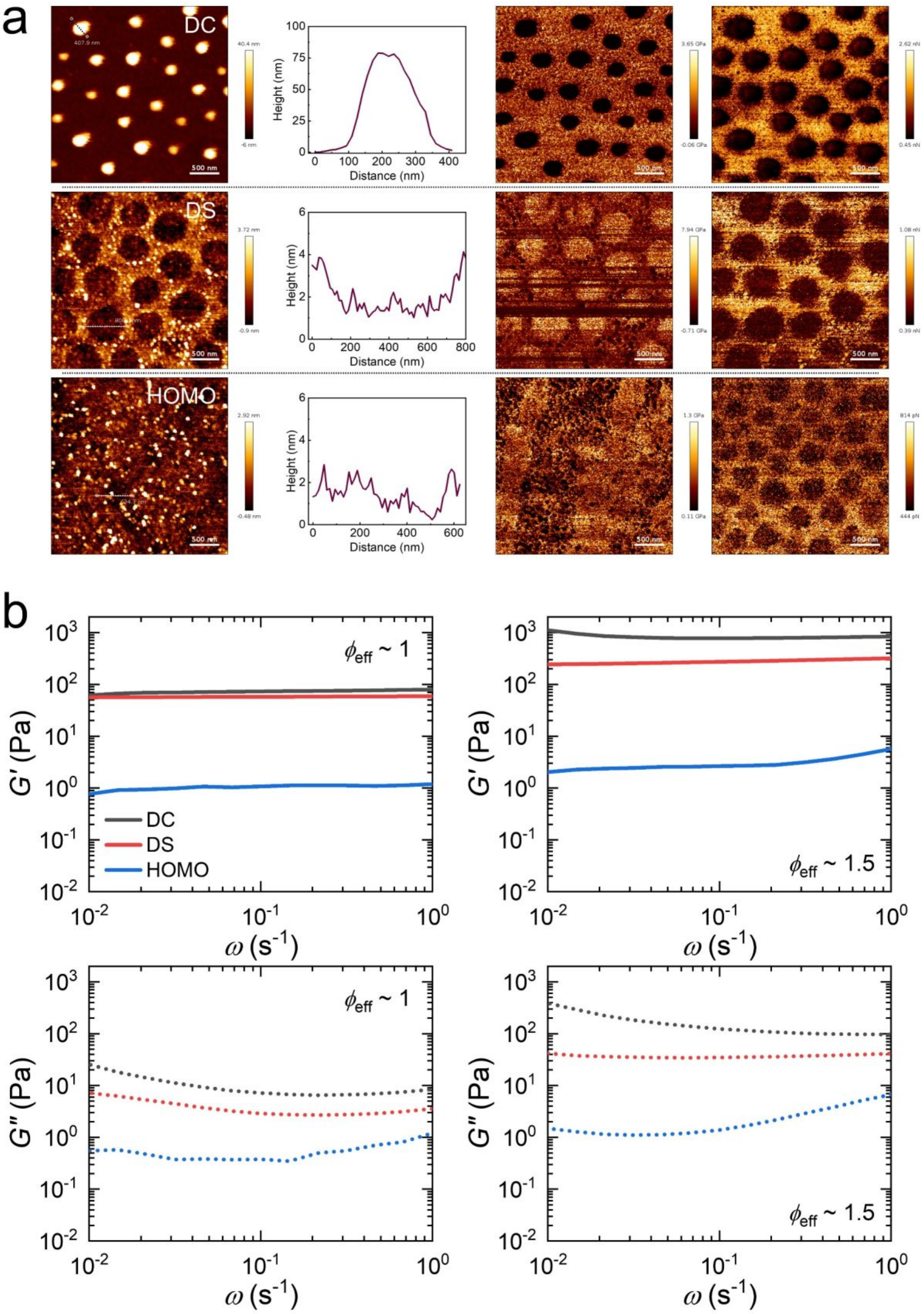

Figure S1. (a) Fluid AFM images of DC, DS, and HOMO microgels adsorbed onto glass substrates in water at 20 °C. From left to right: height, height profile, Young's modulus, and applied force for the vertical approach of the AFM tip toward the solid substrates. (b) Plots of the elastic modulus G' and loss modulus G'' of microgel dispersions at two distinct effective volume fractions ($\phi_{\mathrm{eff}} \sim 1$ and 1.5) of the swollen microgels at 20 °C.

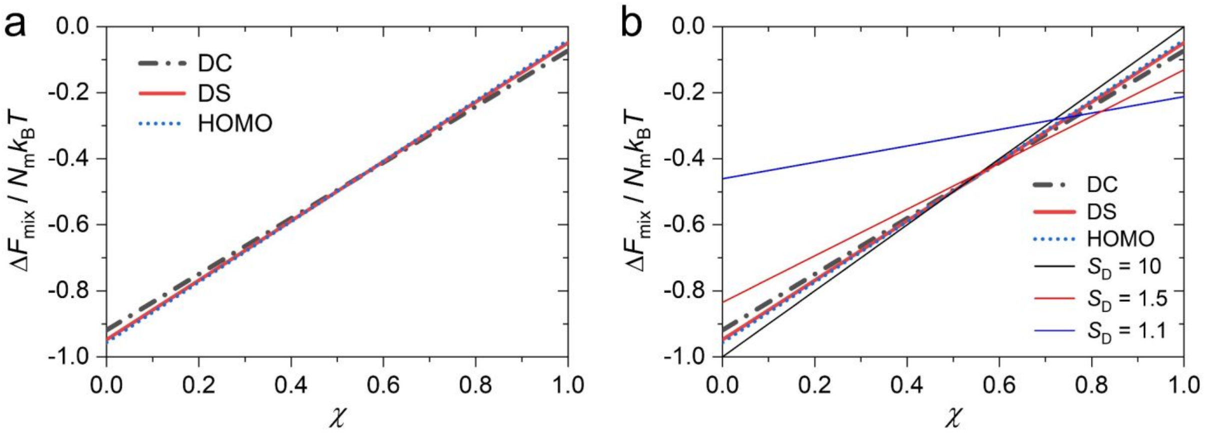

Figure S2. (a) Plots of the mixing free energy ΔF_{mix} as a function of the Flory solvency parameter χ ranging from 0 to 1. (b) Theoretical calculations of the ΔF_{mix} for S_D = 10, 1.5 and 1.1, respectively.

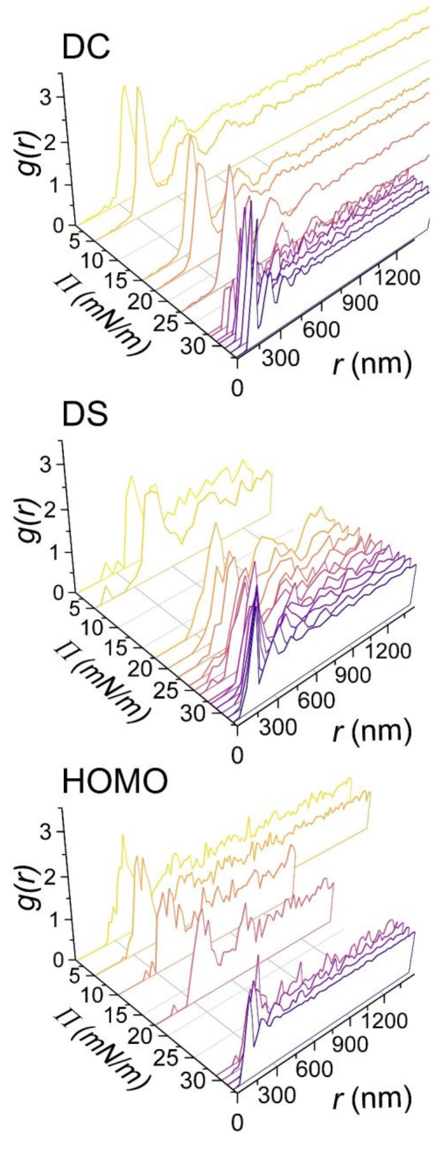

Figure S3. Waterfall plots of the radial distribution function, $g(r)$, for the DC, DS and HOMO microgels as functions of the surface pressure Π and interparticle distance r.

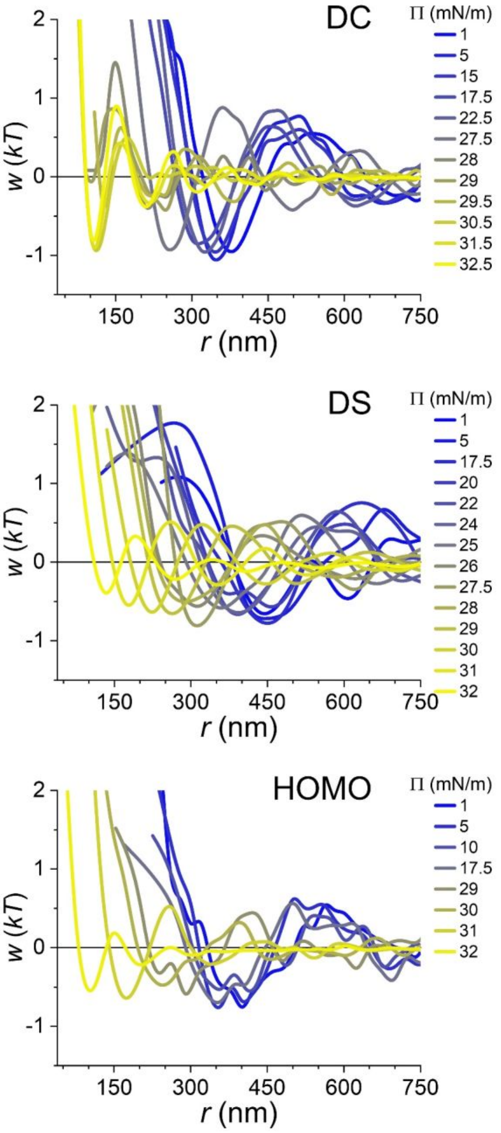

Figure S4. Plots of the interparticle potential profiles $w(r)$ for the DC, DS and HOMO microgels as a function of interparticle distance r at various surface pressure Π.

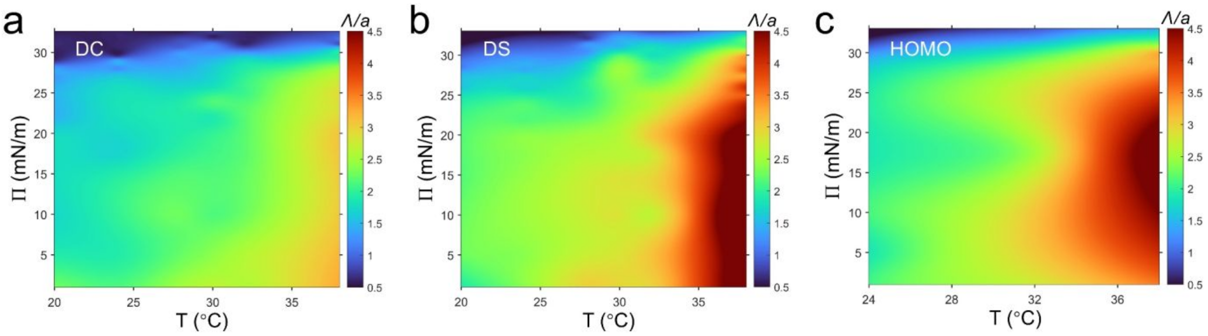

Figure S5. Plots of the nearest neighbor distance, Λ, for the DC (a), DS (b) and HOMO (c) microgels as functions of surface pressure Π and temperature T. Color bars represent the value of Λ/a, where a is the hydration diameter.

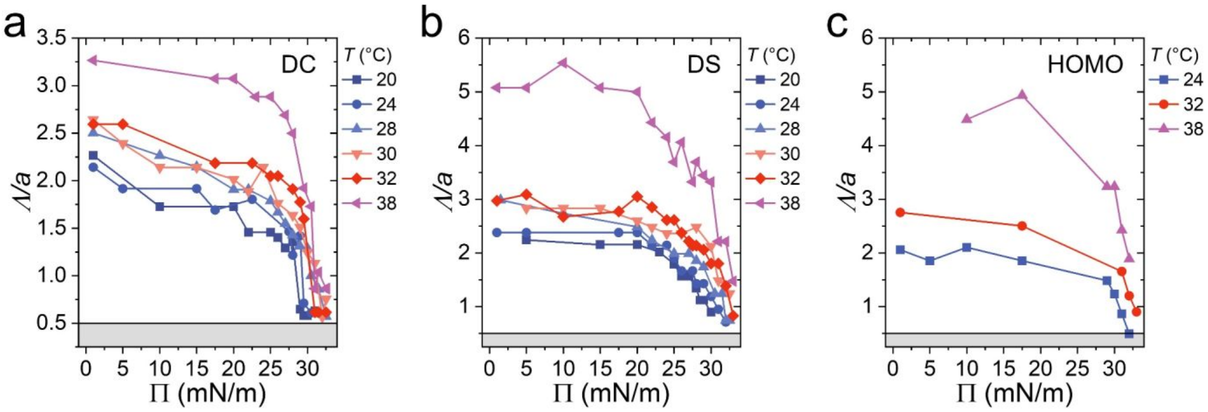

Figure S6. Plots of the nearest neighbor distance Λ for DC (a), DS (b), and HOMO (c) microgels as a function of surface pressure Π at varied temperatures.

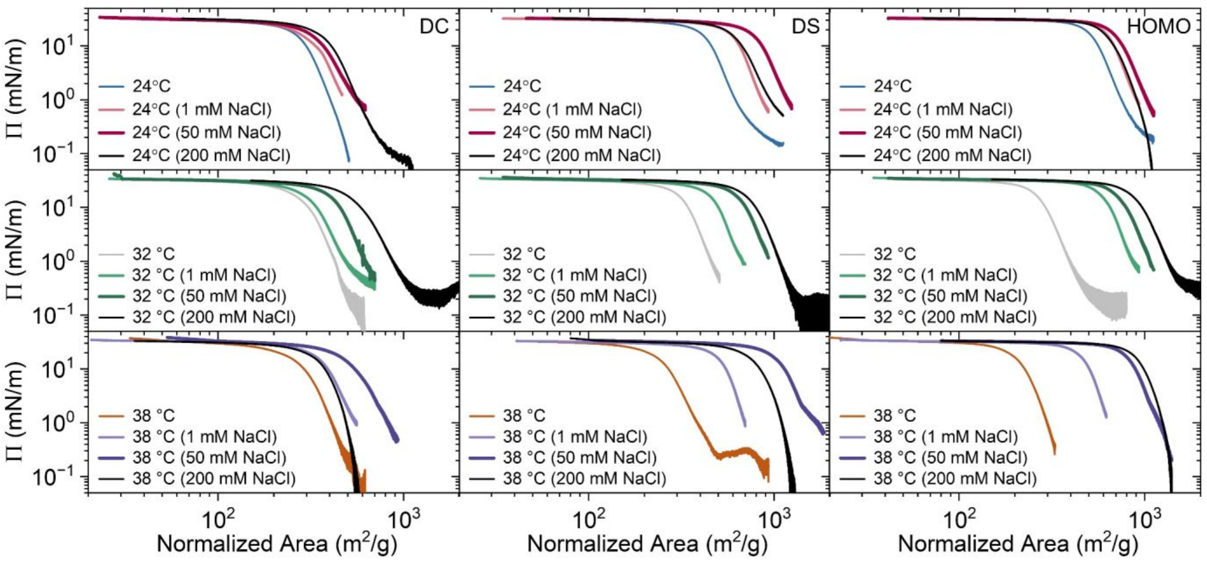

Figure S7. Compression isotherms across the VPTT for the DC, DS and HOMO microgels at varied NaCl concentrations.

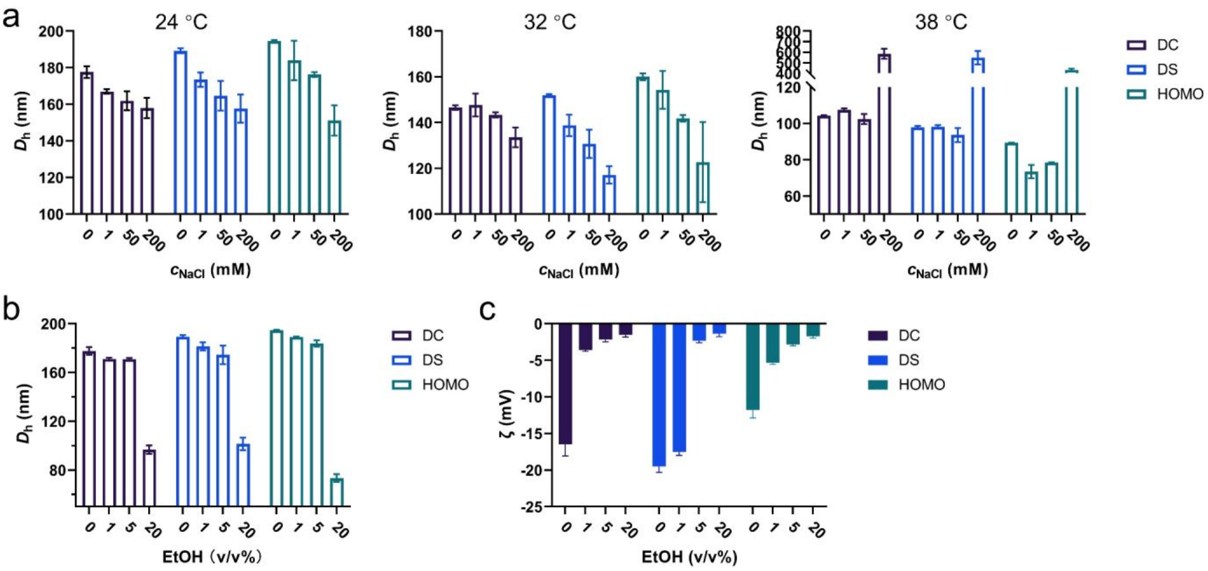

Figure S8. Hydrodynamic diameter (D_h) and zeta potential (ζ) obtained at various conditions.

References

1. Zhou, S.; Chu, B., Synthesis and volume phase transition of poly (methacrylic acid-co-N-isopropylacrylamide) microgel particles in water. *The Journal of Physical Chemistry B* **1998,** *102* (8), 1364-1371.
2. Geisel, K.; Isa, L.; Richtering, W., The Compressibility of pH-Sensitive Microgels at the Oil–Water Interface: Higher Charge Leads to Less Repulsion. *Angewandte Chemie* **2014,** *126* (19), 5005-5009.
3. Han, Z.; Ma, C.; Zhu, H.; Cui, P.; Zuo, T.; Cheng, H., The smearing function for a multi-slit very small angle neutron scattering instrument. *Applied Crystallography* **2024**, *57* (6), 1772-1779.
4. Zuo, T.; Han, Z.; Ma, C.; Xiao, S.; Lin, X.; Li, Y.; Wang, F.; He, Y.; He, Z.; Zhang, J., The multi-slit very small angle neutron scattering instrument at the China Spallation Neutron Source. *Applied Crystallography* **2024,** *57* (2), 380-391.
